# An FPGA-Based Rapid Wheezing Detection System

**DOI:** 10.3390/ijerph110201573

**Published:** 2014-01-29

**Authors:** Bor-Shing Lin, Tian-Shiue Yen

**Affiliations:** 1 Department of Computer Science and Information Engineering, National Taipei University, No. 151, University Road, Sanshia District, New Taipei 23741, Taiwan; 2 Graduate Institute of Biomedical Electronics and Bioinformatics, National Taiwan University, No. 1, Section 4, Roosevelt Road, Taipei 10617, Taiwan; E-Mail: monkeybonjour@gmail.com

**Keywords:** rapid wheezing detection, field-programmable gate array (FPGA), spectrogram image processing, support vector machine (SVM)

## Abstract

Wheezing is often treated as a crucial indicator in the diagnosis of obstructive pulmonary diseases. A rapid wheezing detection system may help physicians to monitor patients over the long-term. In this study, a portable wheezing detection system based on a field-programmable gate array (FPGA) is proposed. This system accelerates wheezing detection, and can be used as either a single-process system, or as an integrated part of another biomedical signal detection system. The system segments sound signals into 2-second units. A short-time Fourier transform was used to determine the relationship between the time and frequency components of wheezing sound data. A spectrogram was processed using 2D bilateral filtering, edge detection, multithreshold image segmentation, morphological image processing, and image labeling, to extract wheezing features according to computerized respiratory sound analysis (CORSA) standards. These features were then used to train the support vector machine (SVM) and build the classification models. The trained model was used to analyze sound data to detect wheezing. The system runs on a Xilinx Virtex-6 FPGA ML605 platform. The experimental results revealed that the system offered excellent wheezing recognition performance (0.912). The detection process can be used with a clock frequency of 51.97 MHz, and is able to perform rapid wheezing classification.

## 1. Introduction

Asthma and chronic obstructive pulmonary disease (COPD) are common around the World. Because of air pollution and other environmental factors, the prevalence of asthma and COPD continues to grow. In 2009, approximately 25 million people in US had asthma, and there were approximately 300 million asthma sufferers worldwide in 2007 [1,2]. Analyzing the spectral density and power of respiratory sounds such as wheezing can yield valuable information. Lung parenchyma and pathological modifications have often been treated as a crucial indicator of asthma and COPD [3].

Current methods of diagnosing asthma include auscultation [4], spirometers, and determining peak expiratory flow to ascertain pulmonary conditions [5]. Conventional stethoscope auscultation is safe and convenient, but also extremely subjective, and cannot be generalized; thus, using auscultation to recognize wheezing is dependent on how experienced the practicing physician is. Although spirometers are used to measure lungs, spirometers induce patient discomfort and are inappropriate for long-term monitoring.

In contrast to traditional manual wheezing detection methods, the use of recording devices to collect and analyze lung sounds has been extensively studied in recent years. The identification of abnormal lung sound characteristics using signal processing methods could help physicians to identify physiological mechanisms generated by lung sounds and their associated pathological links [6]. Because these signal processing methods are objective, their use may also help to establish a classification system to accurately quantify normal and abnormal breath sounds.

It has been medically proven that asthma is a chronic disease from which recovery is not possible. Asthma sufferers have a high risk of suffocation when their asthma is acute, and 250,000 annual deaths are attributed to the disease [2]. Although asthma can be controlled effectively by long-term medication and monitoring, most asthma sufferers are unaware of the condition of their own asthma, and often stop treatment by themselves, causing repeated inflammation and fibrosis in their respiratory tracts, and worsening their lung function. Therefore, the establishment of a portable system for rapid wheezing detection, able to send out a warning during acute asthma attacks, is necessary. Moreover, such a portable system could also be used in home care.

Wheezes are abnormal respiratory sounds that occur for certain duration of time. According to computerized respiratory sound analysis (CORSA) standards [7], wheezing is characterized by its dominant frequency (more than 100 Hz) and duration (more than 100 ms). Most researchers have analyzed wheezes based on spectrograms [8,9,10,11]; this is straightforward, and implementation is simple. However, spectrograms are vulnerable to noise disturbances, and can lack wheezing detection sensitivity. Certain approaches have thus been used to extract wheezing features [12,13,14]; for example, classification models have been combined with algorithms [15,16,17,18], but this requires a large number of coefficients determined through training. This requires immense computational resources, which are not available on portable devices. Another method identified wheezing episodes using image processing [19,20,21] to analyze the edges of spectrograms. However, this method is severely dependent on the resolution of the spectrogram in question. High-resolution spectrograms can be used to improve the sensitivity of this detection system, but also require substantial computational resources.

Improving recognition accuracy thus often requires an immense number of computational resources, and is difficult to implement on portable devices. Therefore, most conventional automatic wheezing detection systems have been built using desktop computers. To implement an automatic wheezing detection system on a portable device, digital signal processors (DSPs) are commonly used [22]. Although DSP has a high clock rate, DSP is inappropriate for wheezing detection because its computation process is based on sequential steps. Another method used a customized integrated circuit (IC) as a DSP coprocessor to detect rapid wheezing; this facilitated hardware acceleration and achieved real-time processing, but involved an immense number of computations. However, a customized IC for rapid wheezing detection is expensive, lacks flexibility, and is unable to be modified or integrated with other systems.

Thus, the field-programmable gate array (FPGA) is ideally suited for building a portable rapid wheezing detection system. Such a portable system can be accelerated by applying an image processing algorithm using parallel computing hardware. The characteristics of wheezes in spectrogram can be treated as quasihorizontal lines with strong amplitude. Thus, there are many image processing techniques combined to preserve these characteristics and filter out unwanted noises. In order to achieve quick response to wheezing events, the frame blocking technique, which divides a spectrogram into sections of two seconds, can reduce responding time and demands of computing resources. Simultaneously, an optimal parameter set for support vector machine (SVM) model proposed in this research shows good accuracy and sensitivity of wheezing recognition. The proposed system was built as an independent wheezing detection silicon intellectual property (WDSIP), able to be integrated with other functional silicon intellectual properties (SIPs), e.g., universal asynchronous receiver/transmitter (UART), direct memory access (DMA), on system-on-programmable-chips (SoPCs) using the peripheral local bus (PLB) and MicroBlaze processor provided by Xilinx. This allowed for greater portability and reduced system volume. In contrast to a customized IC, an FPGA can be modified repeatedly, and can be flexibly integrated with other SIPs. 

## 2. Methodology

### 2.1. Wheezing Detection Algorithm Process Flow

**Figure 1:**
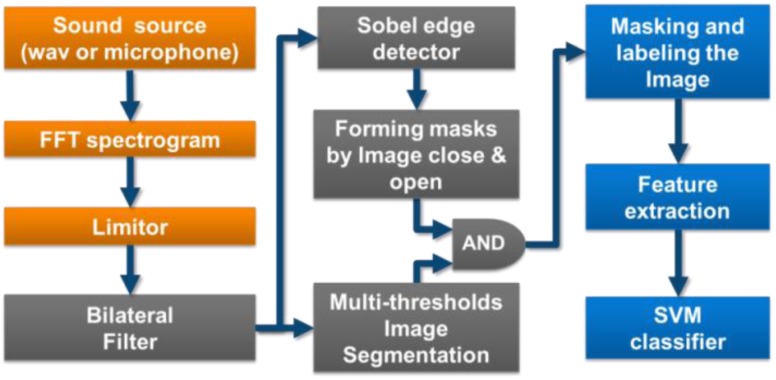
Wheeze detection algorithm processing flow.

The processing flow of our wheezing recognition system is shown in Figure 1, and has three parts:

- Preprocessing: A short-time Fourier transform (STFT) is used to acquire an image containing the time-frequency relationship of the wheezing sound (spectrogram).
- Forming an Image Mask from the Spectrogram: Noise is filtered using a bilateral filter and image processing methods (edge detection, multi-threshold image segmentation, image morphological processing) are used to pinpoint wheezing. A sifting process using two rules based on CORSA standards is also applied to ensure that objects in the image mask are wheezes.
- Feature Extraction and Classification: Features which represent the wheezing components on the masked spectrogram are extracted and classified using an SVM.

Image processing of the spectrogram is the most crucial part of this process. Traditional methods directly analyze the edge of the spectrogram to detect wheezes [19,20], or check peak continuity using rules after the application of image processing techniques (e.g., mean filter) [21]. The proposed system uses a combination of these two methods:

Bilateral filtering is used to both smooth the image by removing outliers, and preserve strong image edge components by giving both spatial and photometric domains weighted coefficients.

Edge detection and multithreshold segmentation are combined to preserve image edges and retain high and isolated peaks during analysis.

### 2.2. SoPC Hardware Architecture

The proposed wheezing detection system was built as an independent WDSIP. The system was built in accordance with SoPC design flow, allowing a number of subsystems to be integrated into a single FPGA, and enabling data transmission between subsystems to be conducted completely on-chip. This reduced I/O speed requirements and additional IC usage of the target platform. This design also allows the system to be used independently or as a part of a broader physiological parameter measurement system.

**Figure 2:**
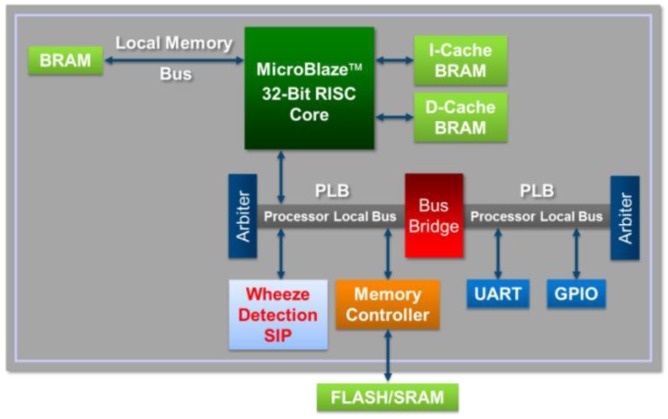
Integrated WDSIP with MicroBlaze processor.

The Embedded Development Kit (EDK) [23] is a software suite provided by Xilinx for designing complete embedded programmable systems. As shown in Figure 2, the EDK allowed a soft processor, MicroBlaze, to be embedded in the proposed WDSIP, allowing the WDSIP to be integrated with other hardware IPs through the PLB. As long as the operational timing of the WDSIP satisfies the requirements of the PLB communication protocol, MicroBlaze can be used to control the setting of the corresponding register on the memory map.

### 2.3. Processor Local Bus (PLB)

The proposed WDSIP requires a PC or a recording device to transfer data through the UART interface (*i.e.*, the ZigBee module). Therefore, the WDSIP was implemented using a PLB interface [24], and MicroBlaze was used to transfer the sound data. The PLB is based on the IBM CoreConnect bus architecture standard for interconnecting MicroBlaze, cores, and custom logic circuitry [25]. The PLB arbiter handles bus arbitration and the transmission of data and control signals between masters and slaves. The output signals of the PLB masters are connected to the PLB arbiter, and the output signals of the PLB slaves are connected to a shared bus back to the PLB through the OR gates. The PLB arbiter thus handles arbitration by multiplexing signals from the masters, which own the PLB bus, onto a shared bus to which all slave inputs are connected. 

**Figure 3:**
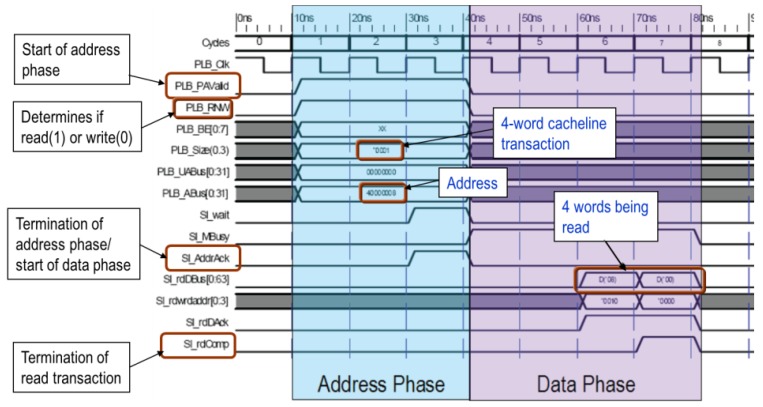
PLB read timing diagram.

As shown in Figure 3, the timing of PLB master and slave communication can be divided into address and data exchange phases. At the start of a transmission, the master is programmed to set PLB_PAValid to “high” to start the address phase. Simultaneously, the master uses PLB_ABus to select a slave to receive the control signal. The communication mode depends on the PLB_Size signal when the slave is receiving or sending data; whether the operation is read or write depends on the PLB_RNW control. After the slave sets the Sl_AddrAck signal to “high,” the data transfer is initiated. After receiving or sending data, the slave sets the Sl_rdComp signal to “high” to confirm the completion of the read or write operation. Table 1 lists the control signals of the PLB interface. 

**Table 1:** PLB interface signals [25].

Signal	Type	Description
PLB_Clk	I	Clock Signal.
PLB_Rst	I	Reset when value = “1”.
PLB_ABus	I	Address, select correspond slave.
PLB_PAValid	I	Address valid, start of the address phase.
PLB_RNW	I	Read when value = “0” and write when “1”.
PLB_BE	O	Specify the number of bytes when PLB_size = “0000”(Single data beat).
PLB_size	O	PLB transfer size, indicates the transfer mode (data width, type, and length).
Sl_addrAck	O	Slave address acknowledge.
Sl_rdBus	O	Slave read data bus.
Sl_rdDAck	O	Slave read data acknowledge.
Sl_rdComp	O	Slave read transfer complete indicator.

### 2.4. Proposed Wheezing Sound Detection System

**Figure 4:**
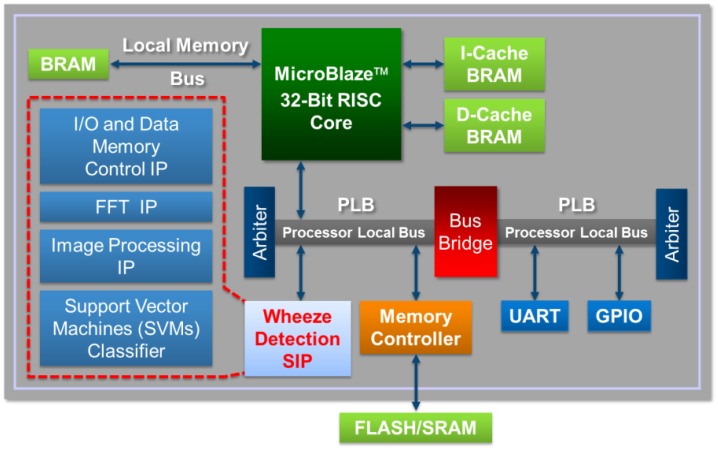
Integrated WDSIP with MicroBlaze (detail).

**Figure 5:**
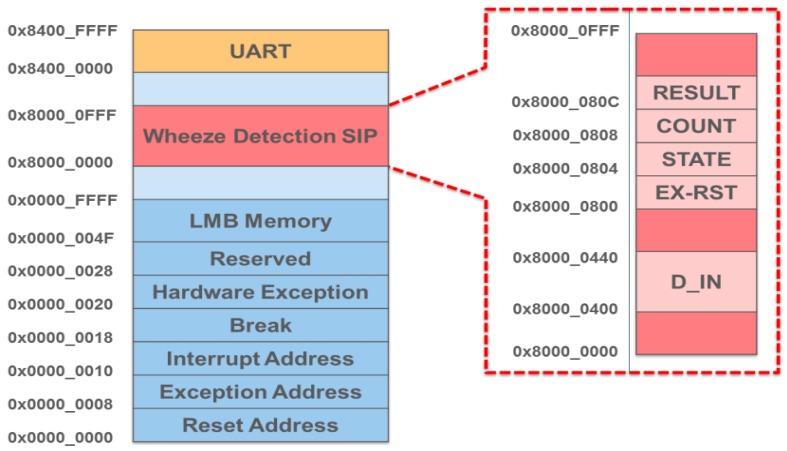
Memory management.

A detailed diagram of the architecture of the system platform is shown in Figure 4. The WDSIP was designed to be able to communicate with other cores through the PLB. As shown in Figure 1, the number of read/write operations necessary during bilateral filtering and image mask formation was determined to be massive. Thus, in the proposed WDSIP, on-chip memory is used to store intermediate data to avoid overusing PLB bandwidth and slowing the processing speed. The WDSIP was designed to use a single PLB slave. MicroBlaze is used only to write the sound data to the WDSIP and read the recognized result from the control register on the memory map. The memory management and function of each register are shown in Figure 5 and Table 2, respectively. Using these control registers, the WDSIP can be controlled using the calling function to read/write the corresponding address on the memory map.

**Table 2:** WDSIP internal register.

Register Name	Direction	Description
EX_RST	I	Reset the wheezes detection SIP.
D_IN	I	Input raw data of breath sound.
STATE	O	Current processing state for MicroBlaze monitoring.
COUNT	O	Counting current input data for MicroBlaze processing bit-error check.
RESULT	I/O	Detection result register, which will be cleared by MicroBlaze when result has been read.

Because MicroBlaze controls the WDSIP using only the reset register, we designed a finite state machine (FSM) to control the internal processing flow (Figure 1). MicroBlaze can only scan the “STATE” register to check whether the FSM has entered the SVM state, and determine whether the current value in the “RESULT” register is valid. After reading a valid value from the “RESULT” register, MicroBlaze clears the “RESULT” register to prevent the future reading of wrong values.

**Figure 6:**
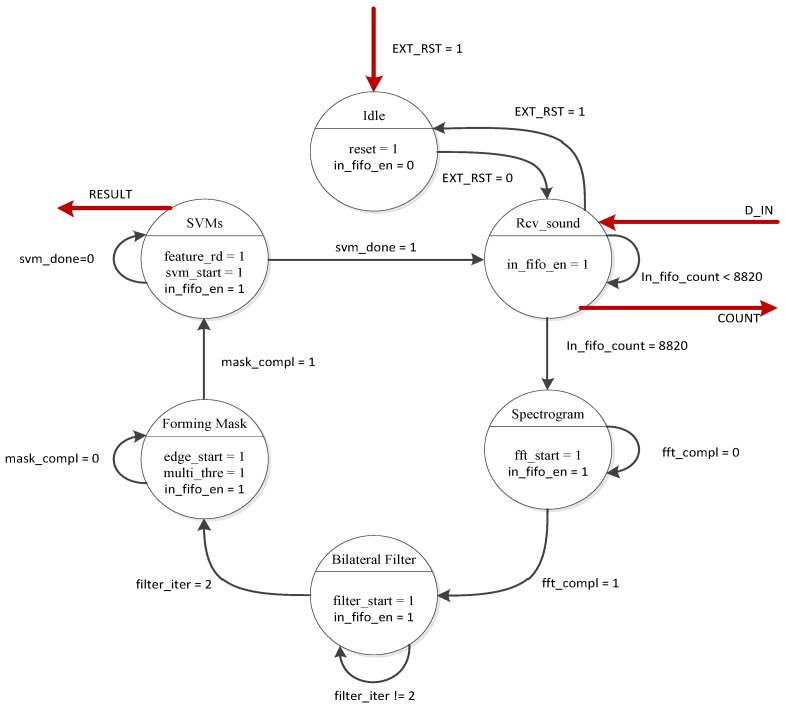
Wheeze detection control finite state machine (FSM).

**Figure 7:**
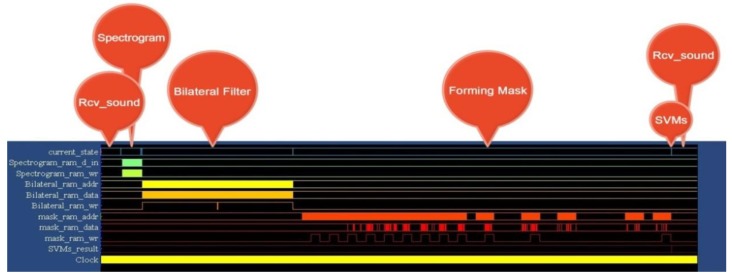
WDSIP timing diagram.

Our control FSM is based on the concept of the Moore machine, and its processing flow is shown in Figure 6. The red arrows represent the registers, which are mapped and controlled by using MicroBlaze, and other arrows represent the internal control signals of the WDSIP. When the external reset signal (EX_RST) is set to “low,” the FSM will enter the Rcv_sound state, and the input FIFO receives external data sent from MicroBlaze (in_fifo_en = 1). The FSM jumps to the next state only when the WDSIP has received a total of 8820 data packets (in_fifo_count = 8820). The input FIFO was designed to be constantly able (in_fifo_en = 1) to receive data, because incoming data may be input at any time to the UART input buffer. The operational timing diagram for the WDSIP is shown in Figure 7.

## 3. Design of WDSIP

Wheezing is characterized by its fundamental frequency and harmonics. Because these characteristics are continuous, spectrograms of wheezing present as quasihorizontal lines that reveal the strong presence of a determined frequency over a period of time. We designed a WDSIP able to rapidly distinguish the distinct edges of these wheeze episodes from background sound components on spectrograms. The main IPs of the WDSIP is described as follows.

### 3.1. STFT Implementation

**Figure 8:**
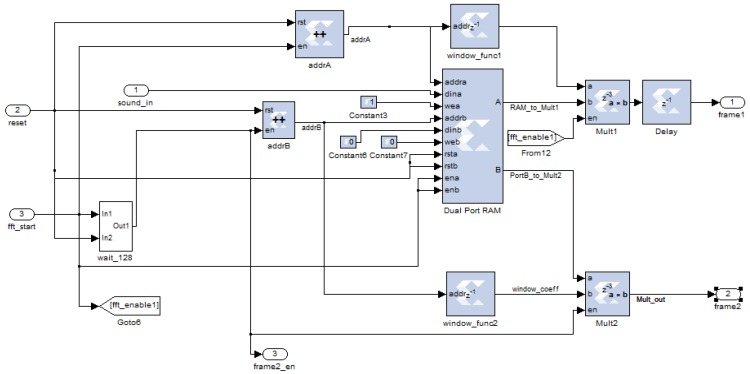
Implementation of 50% overlapping Hanning window.

After the WDSIP collects a frame of sound data, the FSM enters the STFT stage. To implement an STFT with a 50% overlapping Hanning window, the data must be temporary stored in dual-port RAM with a depth of 256. The operational mode of the dual-port RAM was set to read-after-write, allowing the just-stored data to be present on the output port with one cycle delay. This data is multiplied with the weights of the Hanning window to obtain the first data frame. The remaining port waits 128 cycles, then reads the next 128 points, to compute the second data frame, and so on. The actual hardware implementation for the 50% overlapping Hanning window is shown in Figure 8. 

### 3.2. Implementation of the Bilateral Filter

**Figure 9:**
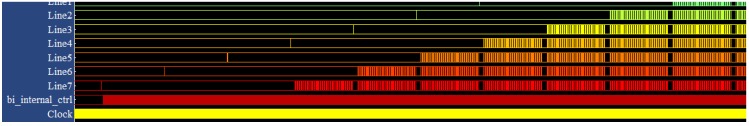
Filling the Masks by Line Buffer.

The bilateral filter implemented in our WDSIP is based on that in [26]. We implemented the image filter in hardware by using a 7 × 7 bilateral filter mask. Thus, to obtain a single filtered central pixel, 48 weights must be computed and multiplied by 48 neighbors in the window of the filter. To avoid a substantial number of computations requiring the use of external memory to store data, a line buffer and register matrix were used to implement the image filter mask. This allowed images to be processed to fill the next pixel at any time. The image mask shifts along the row direction at every pixel clock of the image. All neighborhoods required to be computed in the mask are obtained to calculate the weights in one pixel clock. A filtered image for the central pixel at every pixel clock can thus be obtained. This result could not be obtained using a desktop PC. The timing diagram shown in Figure 9 illustrates the hardware implemented to fill the window of the filter using a delay line. 

**Figure 10:**
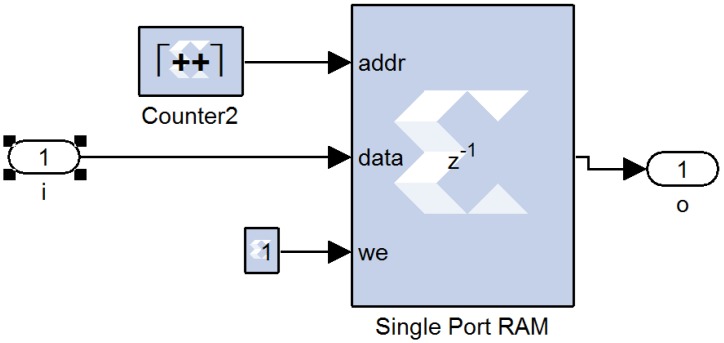
Implementation of the line buffer.

The length of the delay line is related to the size of the image. The delay line was not implemented using shift registers, because processing large image sizes requires substantial hardware resources. The delay line in our system was implemented by using RAM, as shown in Figure 10. We set the depth of the RAM to be equal to the size of image in the row direction, and we set the operational mode to read-before-write. Thus, once a row of data is stored, and the next row of data has entered the RAM, it will first read the data in the RAM, whose column coordinates are the same as the current input data, and write the new data in the RAM to perform the delay line operation. 

Bilateral filter can perform edge-preserving smoothing by applying weights which depend on Euclidean distance and radiometric difference of pixels. In order to reduce the demands on computational resources, the 7 × 7 filter mask can be divided into eight groups because the geometrical location of the mask is radially symmetric. Furthermore, the coefficients of the filter are quantized and stored in the look-up tables (LUTs) to avoid using exponential function. 

### 3.3. Implementation of Multithreshold Image Segmentation

**Figure 11:**
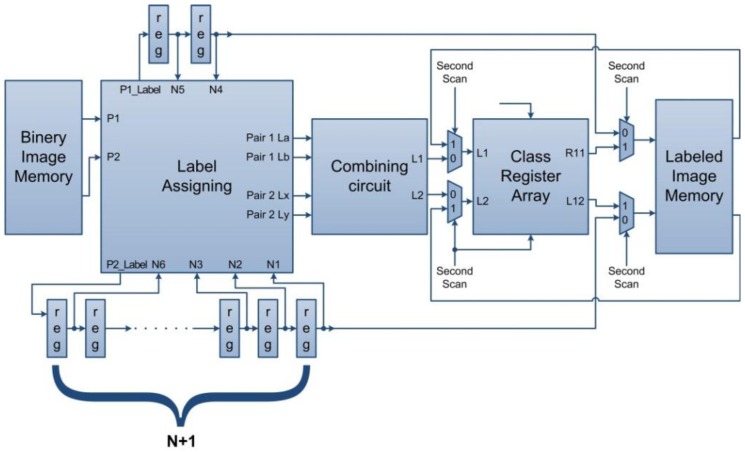
Hardware architecture of image labeling.

In multithreshold image segmentation, a threshold is applied to a filtered spectrogram, an image object on the thresholded image is labeled, and the image objects are isolated using rules. The implementation of the image labeling system in hardware is shown in Figure 11. At first, the content of the class register array is cleared and preset to the initial value. Two pixels (P1 and P2) are then read and thresholded from the filtered image. The pixels are assigned to a label block, then temporarily labeled, and sent to temporary image memory and the delay lines simultaneously, to implement the moving window and perform connectivity checking, as shown in Figure 10. Because the label assigning block may generate two equivalent pairs at the same time, and the class register can only be updated by one equivalent pair at a time, the pairs generated by label assigning block are first processed by a combining block, which rearranges the input order of the labeling pairs, and ensures that they are sent one-by-one. The temporary images are then read from the memory and connected by the content of the class register array. 

**Figure 12:**
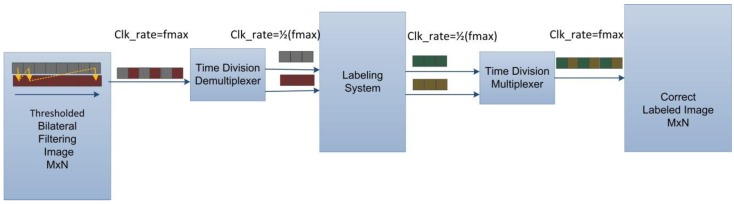
Modified raster scan for labeling system.

In order to isolate all wheezing components, the multithreshold image segmentation, which may require several iterations to apply different thresholds to filter images and check the characteristics of the image objects according to their time and frequency information, becomes the most time-consuming process in the proposed WDSIP. To maximize its speed and use of hardware resources, the solution is adding dual-port RAM and applying pipeline technique to image labeling system to achieve the highest possible clock rate and data throughput. However, only one port of the dual-port RAM can output pixels from the filtered image memory at the highest clock rate, because the other port is at the same time being used to implement the bilateral filter to write the output the filtered image; thus, the operational frequency of this port is 1/8 slower than the highest frequency in the system. As shown in Figure 12, we modified the system to implement a time division demultiplexer, which concurrently sends two pixels to the label assigning block, and returns the output of the labeling system to one-port output using the time division multiplexer. This maintains the processing pixel rate at the system’s maximum speed, and reduces the timing constraint inside the labeling system to half of the maximum speed of the system.

### 3.4. Implementation of Wheezing Mask Formation

**Figure 13:**
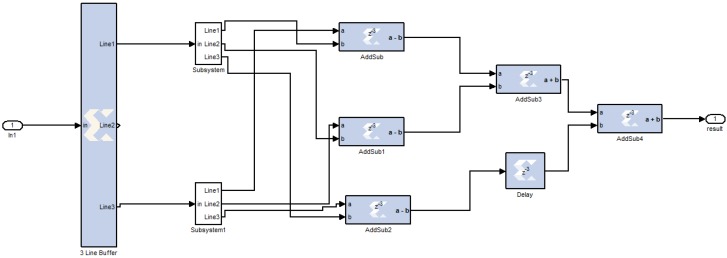
Edge detection processing elements.

Because multithreshold image segmentation only checks the amplitude of the filtered image, many image objects it identifies meet CORSA standards, but not all of them are wheezing components. Edge detection thus extracts quasihorizontal lines with strong gradients from the image. Combining multithreshold image segmentation and edge detection to form an image mask, the spectrogram is masked, and the remaining objects, which are characterized by strong power intensities and strong edges, are considered wheezing components. The hardware implementation of the Prewitt operator for edge detection is shown in Figure 13.

## 4. Results and Discussion

### 4.1. Wheezing Sound Detection Results

**Figure 14:**
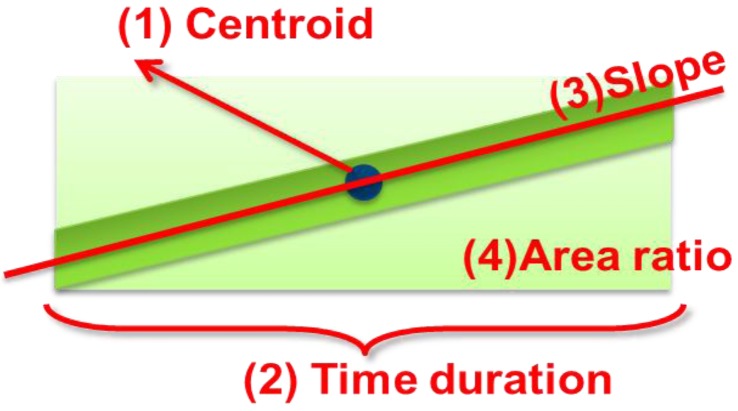
Parameter for extracting wheezing features.

We applied the SVM classifier to predict whether sound data contained wheezes or normal sounds, based on the image properties of the spectrogram objects identified by the mask. As shown in Figure 14, we chose four parameters to extract the wheezing features:

P_CY_: Frequency located at the centroid of the wheezing episode.
P_T_: Time duration of the wheezing episode.
P_S_: Slope of the wheezing episode.
P_AR:_ Area ratio of the wheezing episode/bounding box of the wheezing episode.

These parameters were used to represent the shapes of the objects, and reduce the complexity of the classifier. 

The SVM classifier must be trained for wheezing recognition before implementation. We used the RBF kernel function and grid search method to determine the corresponding *σ* which yielded the most satisfactory result. 

**Figure 15:**
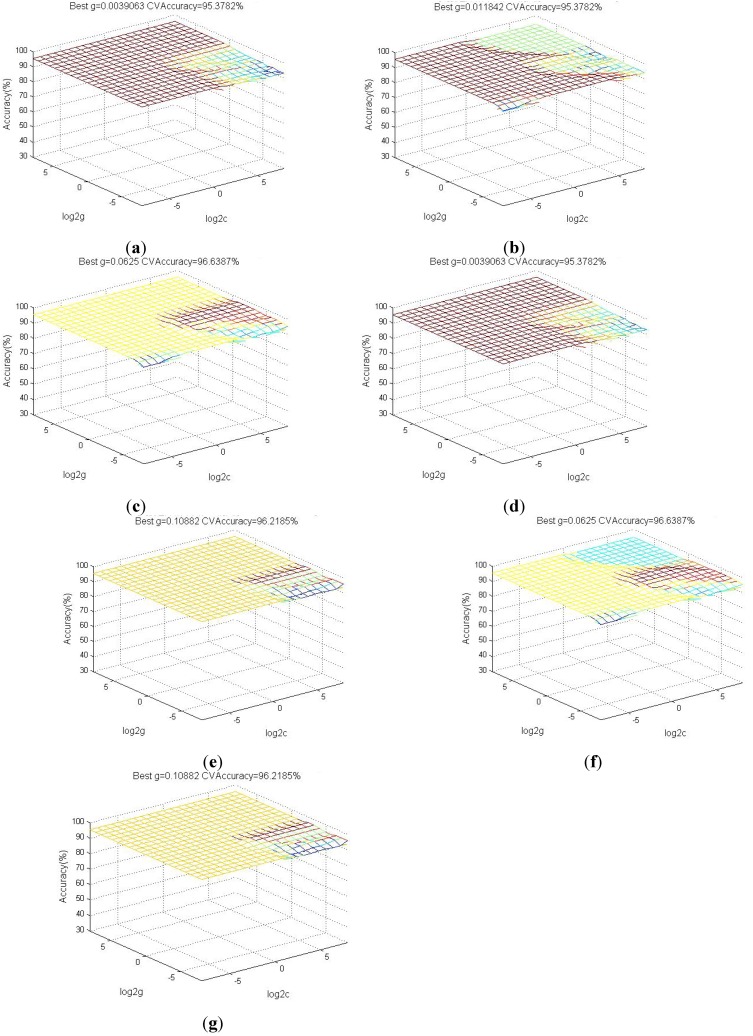
(**a**) Grid searching (Features: P_T_, P_CY_ ); (**b**) Grid searching (Features: P_T_, P_AR_ ); (**c**) Grid searching (Features: P_T,_ P_S_ ); (**d**) Grid searching (Features: P_T_, P_CY_, P_AR_); (**e**) Grid searching (Features: P_T_, P_CY_, P_S_); (**f**) Grid searching (Features: P_T_, P_AR_, P_S_); (**g**) Grid searching (Features: P_T_, P_CY_, P_AR_, P_S_).

Figure 15 show the accuracy of wheezing recognition corresponded to the coefficients of SVM model yielded by using various parameter sets. The performance of the system using various parameter sets was analyzed to verify that the selected parameters adequately assessed wheezing features and derive the most efficient parameter set. We analyzed breath sounds recorded at National Taiwan University Hospital [27], and divided these sounds into training samples and testing samples. The training samples consisted of sound samples recorded from 11 asthmatic patients and 10 healthy people. The testing samples consisted of sound samples recorded from 13 asthmatic patients and 12 healthy people. All sound files were segmented into 2-second units.

The training sample results revealed that the proposed system achieved an accuracy of up to 96.63% using the parameter sets (P_T_, P_S_) and (P_T_, P_AR_, P_S_). After we trained the SVM, we used the trained models to classify the testing samples. The performance (PER) of the recognition system was estimated by calculating the sensitivity (SE) and specificity (SP), defined in the following equations:

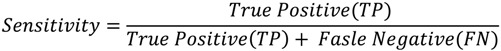
(1)

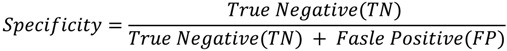
(2)

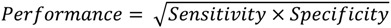
(3)


**Figure 16:**
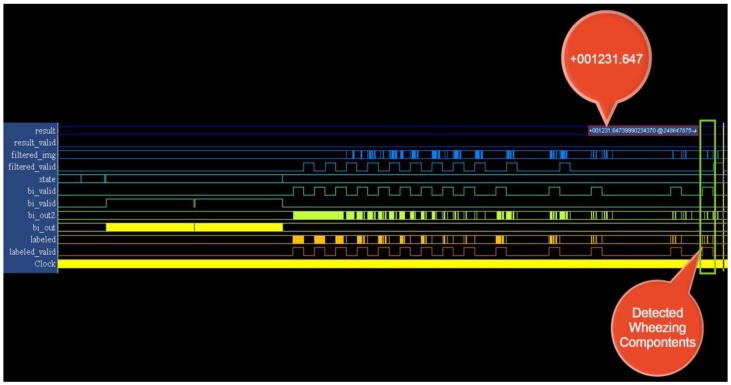
Output of the wheeze detection system (wheezing case).

**Figure 17:**
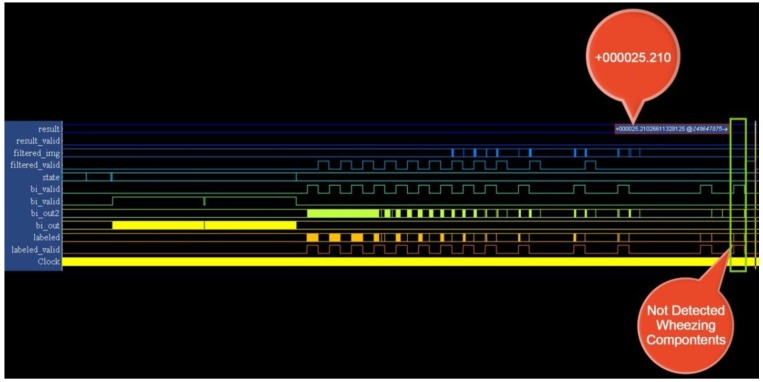
Output of the wheeze detection system (normal case).

The testing samples were analyzed using various parameter sets. The recognition system exhibited superior performance using the parameter sets (P_T_, P_S_) and (P_T_, P_AR_, P_S_), as shown in Table 3. We implemented the SVM model using the parameters (P_T_, P_S_), because these parameters used fewer hardware resources. The results of the implementation of this SVM in hardware are shown in Figure 16 and Figure 17. The system recognizes wheezing episodes when the SVM output exceeds 26.

Identical testing samples were sent to the platform through the UART port to confirm the wheezing detection performance of the WDSIP after the implementation of all functional blocks in hardware. Tera Term was used to connect the platform and set the band rate of serial port to 115,200 bps, to estimate the reliability of the UART transmission from a PC to the platform. We wrote a data set (0–2^32^) to a file, and sent this file to the platform, where a program we had written compared the received data with an accumulator, the estimated error rate of UART transmission was obtained. The results show that no errors were observed when these 4,294,967,296 testing samples were sent to the platform.

As mentioned, fixed-point operation in hardware allows the wheezing recognition error to be predicted. To estimate this error, we assumed that the wheezing recognition results of the software were correct, and compared them with the hardware results. The differences between the software and hardware results are listed in Table 4. The main factor that causes the discrepancy between these results is the depth of the LUT, in which the weight coefficients of the photometric filter are stored. The weight coefficients are quantized, the number of coefficients stored in the LUT is set to 8192, and the precision of the corresponding quantized coefficients is limited to 0.01. This limited precision can be considered the quantization error, which decreases the SNR of the wheezing signal, and impedes the performance of the wheezing recognition system. The performance of the recognition system in hardware and software is compared in Table 5. 

**Table 3:** Recognition results for different features.

Selected Features	TP	TN	FP	FN	Sensitivity	Specificity	Performance
(P_T_, P_CY_)	128	209	21	13	0.907801	0.908696	0.908248
(P_T_, P_AR_)	128	209	21	13	0.907801	0.908696	0.908248
(P_T_, P_S_)	128	215	15	13	0.907801	0.934783	0.921193
(P_T_, P_CY_, P_AR_)	128	209	21	13	0.907801	0.908696	0.908248
(P_T_, P_CY_, P_S_)	124	221	9	17	0.879433	0.96087	0.91925
(P_T_, P_AR_, P_S_)	128	215	15	13	0.907801	0.934783	0.921193
(P_T_, P_CY_, P_AR_, P_S_)	124	221	9	17	0.879433	0.96087	0.91925

**Table 4:** Cross validation.

Cross Validation between Software and Hardware	Amount of Samples
Software classifies as normal and hardware classifies as wheeze	6 samples
Software classifies as wheeze and hardware classifies as normal	11 samples
Total amount of errors	17 samples
Error rate (Total amount of testing samples = 371)	0.0458

**Table 5:** Recognition results from Matlab and hardware.

	TP	TN	FP	FN	Sensitivity	Specificity	Performance
Matlab	128	215	15	13	0.907801	0.934783	0.921193
Hardware	125	216	14	16	0.88652482	0.93913043	0.9124486

As shown in Table 5, the estimated performance of the wheezing recognition system in hardware is impeded because of the quantization error. The wheezing detection procedure is considerably affected by the quantization error because it relies on estimating the gradient by calculating the first derivative of the center pixel. To reduce the effect of the quantization error, the size of the LUT of the photometric coefficients can be increased to increase precision, however, this substantially increases the demand on hardware resources. Therefore, we used a new SVM model based on features extracted from the hardware directly, thus allowing the SVM to yield correct weights.

### 4.2. Implementation Results of the WDSIP

**Figure 18:**
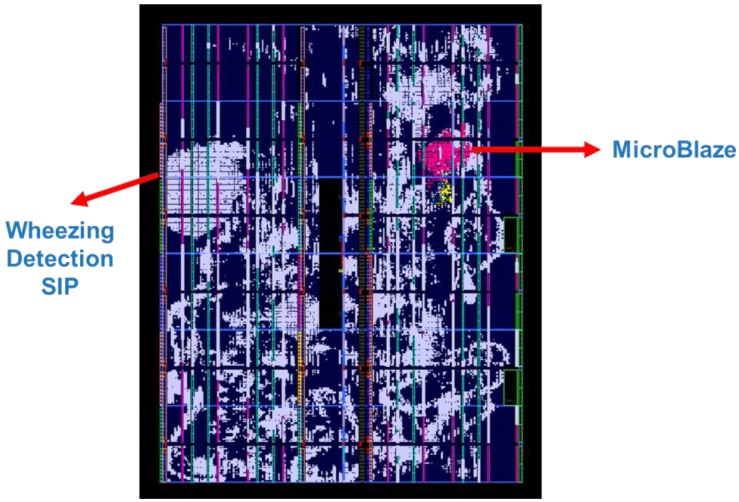
FPGA internal placement and routing.

**Figure 19:**
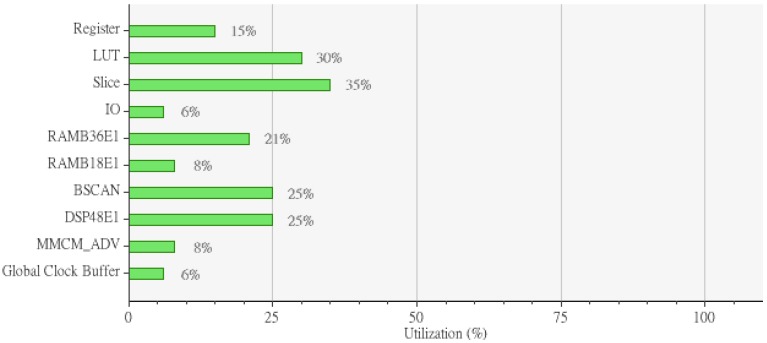
Implemented utilization.

The WDSIP was implemented using a Xilinx Virtex-6 FPGA ML605 platform. The internal placement and routing of the FPGA is illustrated in Figure 18. The total hardware resources used by the WDSIP are listed in Table 6 and Figure 19.

**Table 6:** A summary of the resource usage by WDSIP.

	Spectrogram	Bilateral	Image Labeling System	Morphological	Total
		Filtering		Processing	Used
Slice Register	17511	12881	9228	6449	46069
Occupied Slices	4095	3783	3436	3073	14387
Block RAMs	62	69	48	20	199
DSP Slices	57	66	55	14	192

As shown in Table 6, spectrogram conversion was responsible for using the most hardware resources in the WDSIP implementation. This is because spectrogram conversion requires collecting 8,820 points, and the resulting substantial block RAM demands were not taken into consideration when the depth of the FIFO was set to 8,820. Thus, the FIFO was switched to dual-port RAM to reduce block RAM usage. Moreover, the divider must be used to obtain the power intensities of the spectrogram, and this requires a substantial number of DSP slices to compute. 

We applied a 7 × 7 window when processing the spectrogram by using bilateral filtering, but it is necessary to process more pixels than such a window allows at a time. Although we optimized the architecture of the bilateral filter by dividing the input pixels into eight subgroups, the internal stage of the bilateral filter (*i.e.*, the photometric filter and spatial filter) still required considerable multipliers and adders. Thus, bilateral filtering was found to be responsible for the second-highest hardware resource demands in the system. 

To form the image mask, the temporary image created by the image labeling system after the first raster scanning, as well as the output of the properties scanning system, must be stored in dual-port RAM. To streamline operation and reduce processing time, we implemented additional dual-port RAM, as shown in Figure 12. The system requires three dual-port RAM modules to store these images, requiring a substantial amount of block memory. 

Implemented in hardware, the maximum speed of the WDSIP reached 51.97 MHz; a 2-second breath recording can thus be analyzed for wheezing in 0.07956 seconds. This is adequate for high-speed wheezing detection. We analyzed the power consumption of the WDSIP, and the results are shown in Table 7; the low power consumption of the WDSIP is appropriate for portable device applications. The WDSIP was compared with other portable device applications proposed in previous studies, as shown in Table 8.

**Table 7:** A summary of power consumption of WDSIP.

	Power (W)
Logic Power	0.13203
Signal Power	0.02470
Total	0.15673

**Table 8:** Comparison with previous studies.

	Bahoura [15]	Lin *et al.* [27]	Zhang *et al*. [14]	Yu *et al*. [13]	This Study
Method	GMM + MFCC	MA + BPNN	Sampling	Correlation	Bilateral filter + SVMs
			entropy	coefficient	
Performance	SE= 0.946	SE= 1.0	Not mentioned	SE= 0.83	SE= 0.887
	SP= 0.919	SP= 0.895		SP= 0.86	SP= 0.939
	PER=0.932	PER= 0.946		PER= 0.844	PER= 0.912
Platform	Laptop	Laptop	Laptop and PDA	Laptop and PDA	Standalone
					FPGA system
Speed	Slow	Slow	Fast	Fast	Very Fast

## 5. Conclusions

Wheezing detection systems have mostly been built on desktop PCs. However, these systems are slow and immobile. Especially in home care system, a portable device may reduce the patients’ discomfort. In this study, we designed a portable WDSIP that enables users to analyze wheezing without the use of a PC, which can be feasibly used in remote medical applications. The WDSIP performed at an operational frequency of 51.97 MHz, when implemented using a Xilinx FPGA. The system is able to rapidly perform wheezing detection, which is unachievable using traditional methods. Designed to be compatible with Xilinx PLB and controlled using Xilinx MicroBlaze, the WDSIP offers a high degree of flexibility regarding its potential integration with other biomedical signal detection systems, into more complex SoPCs. Also, the power consumption is a major issue for PC-based wheezing detection system for long-term monitoring. By contrast, implementing WDSIP through advanced complementary metal-oxide-semiconductor (CMOS) process, which reduces the logical and signal power, can perform low power consumption further.

However, the system still has considerable space for improvement: Its hardware must be optimized to reduce demands on hardware resources, and enhance its commercial applicability. Moreover, noise disturbances, and the computational error rate resulting from fixed-point operation, must be more adequately managed. Peripheral devices for the system, such as LCD displays or storage, must also be designed to enable doctors to use the system to diagnose lung diseases, and to allow the system to be implemented in remote medical assistance applications.
